# Adverse Events in Following Immunization with Inactivated SARS-CoV-2 Vaccines (TURKOVAC and CoronaVac) in PCR-Positive Asymptomatic or Mildly Symptomatic Individuals Compared to PCR-Negative Recipients

**DOI:** 10.3390/vaccines14080657

**Published:** 2026-07-27

**Authors:** Ateş Kara, İhsan Ateş, Sebahat Tekcan, Seçil Uysal, Yüksel Hakan Aydoğmuş, Mine Durusu Tanrıöver, Aykut Özdarendeli, Erdogan Oz, Muhammed Emin Demirkol

**Affiliations:** 1 Department of Pediatrics, Pediatric Infectious Disease, Faculty of Medicine, Hacettepe University, Ankara 06100, Türkiye; 2 Turkiye Vaccine Institute, TUSEB Aziz Sancar Arastirma Merkezi, Ankara 06270, Türkiye; 3 Department of Internal Medicine, University of Health Sciences Ankara City Hospital, Ankara 06800, Türkiye; 4 General Directorate of Health Services, Republic of Türkiye Ministry of Health, Ankara 06800, Türkiye; 5 General Directorate of Public Health, Child and Adolescent Health Department, Ministry of Health of the Republic of Türkiye, Ankara 06430, Türkiye; 6 Department of Internal Medicine, Faculty of Medicine, Hacettepe University, Ankara 06230, Türkiye; 7 Vaccine Institute, Hacettepe University, Ankara 06100, Türkiye; 8 Department of Microbiology, Faculty of Medicine, Erciyes University, Kayseri 38039, Türkiye; 9 Vaccine Research, Development and Application Centre (ERAGEM), Erciyes University, Kayseri 38030, Türkiye; 10 General Directorate of Public Health, Ministry of Health of the Republic of Türkiye, Ankara 06430, Türkiye; 11 Department of Internal Medicine, İzzet Baysal Training and Research Hospital, Abant İzzet Baysal University, Bolu 14030, Türkiye

**Keywords:** COVID-19, inactivated vaccine, adverse event, asymptomatic, mild symptoms, TURKOVAC

## Abstract

**Background/Objectives**: COVID-19 vaccines may be given without requiring a negative test or absence of symptoms; however, this recommendation needs further safety evaluation. This study aimed to compare the adverse events following immunization (AEFI) between PCR-positive (asymptomatic/mildly symptomatic) and PCR-negative individuals at the time of vaccination, with a secondary objective of comparing the two vaccines (TURKOVAC and CoronaVac). **Methods**: This descriptive study was a secondary analysis of phase III clinical trials. Individuals who were positive for SARS-CoV-2 PCR at the time of vaccination constituted the PCR(+) group and those who tested negative or became positive after the 4th day following vaccination constituted the PCR(−) group. Whether participants had a history of COVID-19 was not considered. **Results**: Data from 5207 individuals were analyzed. AEFIs were more frequent in the PCR(+) group than PCR(−) group: site pain (24.5% vs. 16.7%, *p* < 0.001), arm pain (9.5% vs. 7.8%, *p* = 0.008), headache (14.8% vs. 10.0%, *p* < 0.001), fatigue (14.5% vs. 8.9%, *p* < 0.001), myalgia (12.3% vs. 7.5%, *p* < 0.001), sore throat (11.9% vs. 8.1%, *p* < 0.001), cough (7.9%; 11.1% vs. 6.9%, *p* < 0.001). These AEFIs were more frequent in the PCR(+) group with both vaccine groups separately. The distribution of AEFIs over time followed similar patterns in PCR(+) and PCR(−) individuals. Local AEFIs were slightly more common with the TURKOVAC, systemic AEFIs with the CoronaVac. **Conclusions**: While AEFIs were more common among PCR(+) group, they were mild, tolerable, and easily manageable. Our results support the suggestion that routine PCR testing prior to vaccination may not be warranted solely to mitigate concerns about anticipated adverse events.

## 1. Introduction

It has been 7 years since the first reported cases of several atypical pneumonia cases in December 2019 in Wuhan, China, which was then identified to be caused by a novel coronavirus named Severe Acute Respiratory Syndrome Coronavirus 2 (SARS-CoV-2). The spread rapidly evolved into the most significant public health emergency of the century—the COVID-19 pandemic. By the first days of 2026, 779 million cases and 7 million deaths had been reported worldwide by the World Health Organization (WHO), and Türkiye is one of the countries with the highest disease burden, which COVID-19 accounted for 17 million cases and 101,000 deaths [1]. Currently, the majority of the cases are now asymptomatic or mild, but it still poses a significant risk for vulnerable populations, highlighting the importance of continued prevention measures. Widespread vaccination has been the most critical protective strategy, and as of January 2025, there are 50 approved vaccines worldwide, 12 of which were granted emergency use listing by the WHO [2]. The COVID-19 vaccines are grouped under two main categories: those with viral component vaccines and the whole-virus vaccines. RNA-based COVID-19 vaccines are classified as viral component vaccines, whereas inactivated vaccines are whole-virus vaccines and contain copies of the SARS-CoV-2 virus that have been inactivated. In Türkiye, the mRNA vaccine Comirnaty, adenovirus vector-based vaccine Sputnik V (Gamaleya), CoronaVac, and the TURKOVAC were approved for use during the pandemic. Among these vaccines, TURKOVAC and CoronaVac are whole virion-inactivated vaccines. Previous Phase I and Phase II studies reported the safety and immunogenicity profile and the dose effectiveness of TURKOVAC [3], and a subsequent phase III trial showed that two-dose schedules of TURKOVAC were safe and tolerable, and the efficacy was non-inferior to CoronaVac (Sinovac Life Sciences Co., Ltd., Beijing, China) [4].

Currently, the Centers for Disease Control and Prevention (CDC) [5] and WHO [6] do not recommend serologic testing to evaluate the protection against COVID-19 or the current disease status before vaccination. Recommendations also include that everyone should take vaccines even if there is a history of COVID-19 unless the individuals have a history of severe allergic reactions/anaphylaxis to any of the ingredients of the COVID-19 vaccine or have a fever over 38.5 °C on the day of vaccination. Moreover, there is no recommendation about not getting vaccinated in the presence of mild symptoms related to the clinical rationale, although it is not recommended due to protecting others and healthcare personnel from the spread of the disease [7]. Per the recommendations from the international authorities, Türkiye applied similar procedures, and a negative test result or being asymptomatic at the time of vaccination was not required to get the vaccines. However, this does not rule out the safety concerns with this policy.

Although the COVID-19 vaccines are highly effective in preventing the disease, they are not without risk of developing adverse events following immunization (AEFIs), and the WHO defines AEFIs as medical events that follow vaccination and that do not necessarily have a causal relationship with the use of the vaccine [8]. With particular regard to the unprecedented rapid development and mass deployment of COVID-19 vaccines, evaluating the AEFIs is crucial to establishing the safety profiles of the vaccines. It may not always be clear to distinguish between vaccine-related AEFIs and the adverse events of another cause like comorbidities, etc.; analyzing existing data from vaccine trials may make this differentiation possible and is crucial to monitor the safety of the products.

COVID-19 infections may manifest without symptoms or exhibit only minimal clinical findings. Research indicates that around 30–40% of all infections could be asymptomatic, with even greater prevalence observed in specific outbreak contexts [9,10,11]. In this study, we evaluated whether the administration of an inactivated COVID-19 vaccine (TURKOVAC and CoronaVac) to individuals who are asymptomatic or with minimal symptoms results in different adverse effects compared to those with a documented absence of prior infection. By addressing this question, we aimed to provide evidence that could assist vaccine practices in addressing potential concerns and enhance confidence in vaccine administration.

Based on this background, this study aimed to evaluate the AEFIs in individuals who were asymptomatic or had mild symptoms at the time of vaccination in a secondary analysis of data from the previous clinical trials of vaccines (TURKOVAC and CoronaVac) to demonstrate the adequacy of symptom inquiry during patient interviews for vaccine administration. For this purpose, AEFIs were compared between individuals who tested positive for SARS-CoV-2 PCR (asymptomatic/mildly symptomatic) at the time of vaccination and those who tested negative or positive after the 4th day following vaccination. The frequency of AEFIs was also compared between the two vaccines (TURKOVAC and CoronaVac).

## 2. Materials and Methods

This descriptive study was designed as a secondary analysis of data from two phase III clinical trials of TURKOVAC. The first study (ClinicalTrials.gov ID: NCT04942405) [12] was a randomized, observer-blinded phase III clinical trial conducted to evaluate the efficiency, immunogenicity, and safety of the two-dose inactivated COVID-19 vaccine (TURKOVAC) versus the two-dose CoronaVac (Sinovac) vaccine in healthy subjects and the second study (ClinicalTrials.gov ID: NCT05077176) [13] was an open-label, multicenter phase III clinical trial to evaluate the efficiency, safety, and immunogenicity of booster dose application of inactivated COVID-19 vaccine (TURKOVAC) after the second dose of CoronaVac (Sinovac). The data from these studies were aggregated for descriptive analyses. The participants’ symptom status was evaluated clinically at enrollment, and since individuals with fever and prominent respiratory symptoms were excluded, the participants included had only clinically mild-to-moderate local and respiratory symptoms. The protocol of the NCT04942405 study was approved by the Clinical Research Ethics Board of Hacettepe University (Approval No: KA-21070 and Date: 21 June 2021)—the protocol of which was published elsewhere [4]. The protocol of the NCT05077176 study was approved by the Clinical Research Ethics Committee of Ankara City Hospital (Approval No: E2-21-889 and Date: 5 October 2021). Informed consent was obtained from all volunteers involved in both studies.

The primary objective was evaluating the AEFIs in individuals who tested positive for SARS-CoV-2 PCR at the time of vaccination (PCR+ group) and in individuals who tested negative or tested positive after the 4th day of immunization (PCR− group). Whether participants had a history of COVID-19 was not considered. In each study group, the local and systemic AEFIs were compared between patients who received TURKOVAC or CoronaVac in the original clinical trials. The severity of clinical AEFIs was assessed in the original trials using Food and Drug Administration (FDA) toxicity grading scales for vaccine clinical trials [4]. Accordingly, mild AEFIs (Grade 1) were defined as transient (<48 h) or mild discomfort that no medical intervention/therapy required, and moderate AEFIs (Grade 2) as mild-to-moderate limitation in activity—some assistance may be needed with no or minimal medical intervention/therapy required. The AEFIs are also categorized over time after vaccination as the events seen in 30 min, 24 h, 3-day, and 7-day periods (assessed cumulatively) after vaccine administration, as described in the original phase studies and published previously [3].

In this study, pooled data from two studies were used. NCT04942405 was a two-dose primary-series study, while NCT05077176 was a booster study. We are aware that reactivity and AEFI profiles may differ between primary-series and booster vaccinations because of secondary immune responses. A comparative table of local and systemic adverse events from both studies is provided in the Supplementary Materials (Table S1). There were statistically significant differences in the frequency of AEFIs between the primary-series and booster trials for nearly all local and systemic events. However, despite this heterogeneity, we used pooled data because no serious vaccine-related adverse events (grade 4 or 5) were observed in either study, and the primary aim of our study was to compare AEFI profile between PCR-positive and PCR-negative groups.

### 2.1. PCR Methodology

The PCR methodology used in the study registered under ClinicalTrials.gov ID of NCT04942405 was described in detail by Tanriover et al. [4]. In brief, oropharyngeal and nasopharyngeal swabs were collected from all participants at the first visit 1 for baseline PCR testing with Bio-Speedy^®^Direct RT-qPCR SARS-CoV-2 detection kit (Bioeksen, Istanbul, Türkiye), performed on Bio-Rad CFX96 TouchTM platform (Hercules, CA, USA). For the study registered under ClinicalTrials.gov ID of NCT05077176, PCR testing was conducted independently by each participating center in accordance with their institutional protocols. A positive PCR test indicates current infection with SARS-CoV-2.

### 2.2. Statistical Analyses

Descriptive data for AEFIs in vaccine groups were presented using frequency and percent values, and the categorical data were compared between independent groups using the Chi-square test or Fisher’s exact test if assumptions for a Chi-square test were not met. To determine the factors influencing the occurrence of AEFIs, a logistic regression model including sex, age, PCR results and administered vaccines as independent variables was analyzed using the Enter method. A two-sided *p*-value < 0.05 (type-I error level of 5%) was considered statistically significant. All analyses were made in SPSS Version 29 (IBM SPSS Statistics for Windows, Armonk, NY, USA: IBM Corp).

## 3. Results

**Figure 1:**
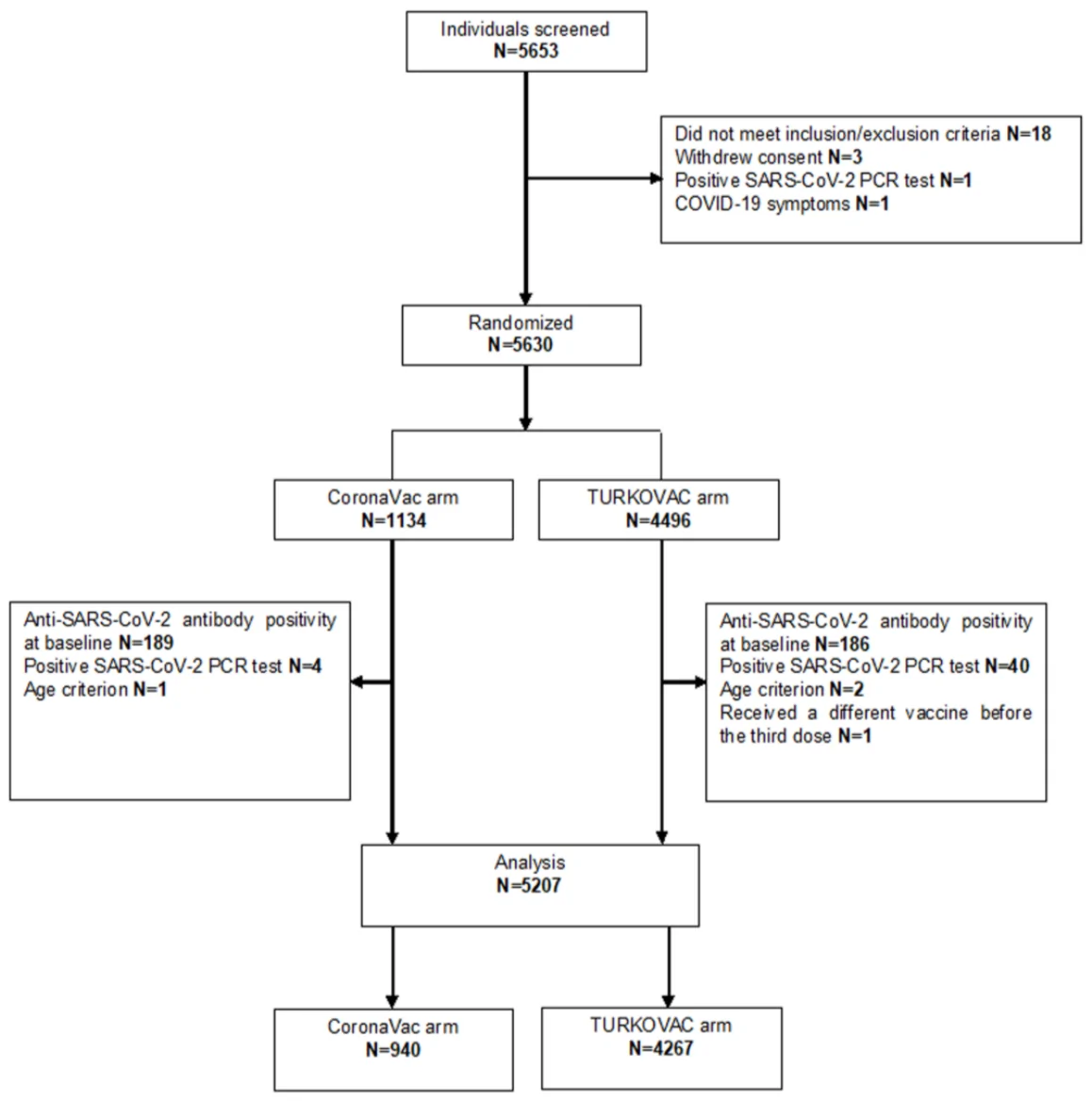
Study flow chart.

The aggregated data from the reference clinical trials included a total of 5207 individuals. TURKOVAC was administered to 4267 volunteers, and CoronaVac to 940. Study flow chart has been presented in Figure 1. Of the vaccinated individuals, 4021 were in the PCR(+) group and 1186 were in the PCR(−). The PCR(−) group was defined as those who tested negative or positive from the 4th day after vaccination; however, no cases of PCR tests turning positive were observed in the PCR(−) group during the post-vaccination side effect monitoring period.

Demographic characteristics of participants are presented in Table 1. PCR-positive participants were younger than PCR-negative participants, with statistically significant differences observed in both vaccine groups (CoronaVac: 39 ± 9 vs. 41 ± 10 years, *p* = 0.007; TURKOVAC: 41 ± 9 vs. 43 ± 9 years, *p* ≤ 0.001). Sex distribution did not differ significantly between PCR-positive and PCR-negative individuals in the CoronaVac group (*p* = 0.290); however, it differed in the TURKOVAC group (*p* = 0.001), with a predominance of male participants in both groups.

The most common local AEFIs identified were pain in the injection site (18.5%), pain in the arm (8.6%), swelling/nodules in the injection site (4.7%), and erythema in the injection site (1.3%). Local AEFIs were seen in slightly higher proportions in the TURKOVAC group. The most common systemic AEFIs were headache (11.1%), fatigue (10.2%), myalgia (8.6%), sore throat (8.9%), and cough (7.9%), and all but myalgia seen in higher proportions in the CoronaVac group. Table 2 summarizes the breakdown of the AEFIs in the vaccine groups and both groups combined.

The distribution of AEFIs in the PCR(+) and PCR(−) groups is presented in Table 3. Pain in the injection site (*p* < 0.001) and pain in the arm (*p* = 0.008) were the only significantly different local AEFIs, which were seen more frequently among the PCR(+) group. However, all systemic AEFIs were significantly more frequent in the PCR(+) group.

When the distribution of AEFIs in PCR(+) and PCR(−) groups is evaluated separately for the two vaccines, TURKOVAC group revealed that pain in the injection site (*p* < 0.001) and pain in the arm (*p* = 0.002) were the only significantly different local AEFIs, which were seen more frequently among the PCR(+) group. In the CoronaVac group pain in the injection site was significantly more frequent among local AEFIs (*p* = 0.020). All systemic AEFIs were more frequent in the PCR(+) group in the both vaccine group. The data are shown in Table S2 in the Supplementary Materials.

The distribution of local and systemic AEFIs across different time points in PCR(+) and PCR(−) groups was presented in Table 4. There was no significant difference between the PCR(+) and PCR(−) groups in terms of the frequency of local and systemic AEFIs at 30 min. All AEFIs in the following time period were more frequent in the PCR(+) group.

The proportion of events seen in 30 min of administration in the PCR(+) group was similar between the vaccine groups (*p* = 0.69), and only 0.8% of individuals in each vaccine group had AEFI. In the PCR(−) group, AEFIs were more frequent in CoronaVac (1.3% vs. 0.4%, *p* = 0.014), mainly local AEFIs.

The aggregated AEFIs seen 24 h after vaccination were not significantly different between TURKOVAC and CoronaVac in both the PCR(+) (*p* = 0.94) and PCR(−) groups. But disaggregated distributions showed local AEFIs were more frequent in the TURKOVAC group in PCR(+) (22.6% vs. 14.7%, *p* = 0.007) and PCR(−) (15.3% vs. 11.7%, *p* = 0.019) individuals. Also, systemic AEFIs were more frequent in the CoronaVac group (14.3% vs. 5.4%, *p* < 0.001) in PCR(+) individuals.

The AEFIs showed similar distribution patterns in the 3 days. The TURKOVAC group had more events in both PCR groups (PCR(+): 47.8% vs. 40%, *p* = 0.030; PCR(−): 34.3% vs. 25.2%, *p* < 0.001). The breakdown also showed that local AEFIs were more frequently seen in TURKOVAC recipients in both PCR groups (*p* < 0.001). In contrast, systemic AEFIs were more frequent in CoronaVac in PCR(+) individuals (*p* < 0.001).

The 7-day interval after the vaccination also had a similar distribution for AEFIs. Local events were more frequent among TURKOVAC recipients in both PCR(+) (41.4% vs. 20%, *p* < 0.001) and PCR(−) (28.9 vs. 16.9%, *p* < 0.001) groups and systemic AEFIs were more frequent in CoronaVac recipients (32.8% vs. 21.3%, *p* < 0.001) in the PCR(+) group. Supplementary Table S3 provides detailed data.

In the logistic regression analysis (Table 5), female sex, PCR positivity, and TURKOVAC administration were identified as increasing factors for the overall AEFIs and local AEFIs, while advancing age was associated with reduced odds. Notably, in the systemic AEFI model, TURKOVAC was associated with significantly lower odds. Female sex and PCR positivity remained significant predictors of systemic AEFIs, whereas increasing age showed protective effect. These findings indicate that while TURKOVAC is associated with a higher incidence of local reactions, it demonstrates a comparable or potentially favorable systemic safety profile relative to CoronaVac.

## 4. Discussion

This descriptive, secondary analysis of the previous phase III clinical trials of TURKOVAC showed that adverse events seen in the immediate and short term after TURKOVAC and CoronaVac vaccine administrations had similar distribution patterns between PCR-positive and negative groups. However, regardless of the timing of the emergence of the AEFIs, the pain in the vaccination site and arm and the systemic AEFIs were more frequently observed in the PCR(+) groups. This observation may reflect increased vaccine reactogenicity; however, in the absence of supporting immunological data, it remains challenging to determine whether systemic AEFIs are more frequently associated with vaccine reactogenicity or with COVID-19 infection. Local AEFIs were more frequent in the TURKOVAC group, whereas systemic AEFIs were more frequent in the CoronaVac group. All AEFIs were more frequent in PCR-positive individuals; however, both local and systemic events were mild and well tolerated. These results are suggestive that the administration of both vaccines can be considered safe in individuals even if they have COVID-19 but are asymptomatic or have mild symptoms at the time of vaccination, and TURKOVAC is associated more with milder local AEFIs compared to its comparator, CoronaVac, in the immediate and short term after administration.

The safety of the TURKOVAC vaccine in phase III clinical trials was published previously. Tanriover et al. [4] reported in their interim analysis of the randomized, observer-blinded, non-inferiority phase III study for efficacy, immunogenicity, and safety of the two-dose schedules of TURKOVAC versus CoronaVac that TURKOVAC was safe and non-inferior to its comparator and provided a relative risk reduction of 41.03% after 14 days of the second dose. Also, safety analyses among individuals who received at least one dose showed total AEFIs, including both local and systemic events, were more frequent in the TURKOVAC arm, but none were severe AEFIs. Another recent study by Tanriover et al. [14] also evaluated the impact of previous SARS-CoV-2 infection on post-vaccine adverse events in individuals vaccinated with inactivated COVID-19 vaccines. The secondary analysis of data from the randomized, observer-blinded, non-inferiority study of TURKOVAC revealed that the frequencies of local and systemic AEFIs after the second dose were similar between those with and without a COVID-19 history and the TURKOVAC and CoronaVac showed similar frequencies of local and systemic AEFIs in the first 30 minutes after vaccination. The added value of our current study is evaluating the AEFIs in patients who were asymptomatic or had mild symptoms at the time of vaccination comparatively to individuals without the disease, and our results showed that TURKOVAC was also safe to administer in the presence of mild COVID-19 disease. Also, the similarity in the distribution patterns of AEFIs between individuals with and without COVID-19 suggested that molecular testing is not needed to evaluate the disease status before vaccine administration, which is also per the recommendations of the international authorities.

The global actions for widespread and population-based vaccination approaches have reached 67% of the world’s population vaccinated with a complete primary-series of a COVID-19 vaccine and 32% of the population vaccinated with at least one booster dose [1]. As of July 2026, 93% of the adult population in Türkiye has received at least one dose of COVID-19 vaccine, while 86% has completed two doses [15]. These large-scale vaccination policies have provided incomparable benefits regarding mortality and morbidity against COVID-19 and also resulted in widespread immunity across populations. However, as the disease evolves, novel SARS-CoV-2 genetic variants have emerged, increasing transmissibility and evading host immunity [16]. The WHO Strategic Advisory Group of Experts on Immunization (SAGE) also addressed the evolving public health needs as the Omicron variant and its sub-lineages continue circulating and recommended updates for simplified vaccine schedules for programmatic COVID-19 vaccinations [17]. Moreover, the decreasing seropositivity in the population over time and the changing characteristics of novel variants resulted in higher proportions of cases with asymptomatic infections or mild symptoms [18]. Given these changes in disease characteristics, our results also suggest that more prevalently seen asymptomatic or mildly symptomatic individuals can get the TURKOVAC vaccine without needing a serologic assessment based on its safety profile.

To the best of our knowledge, this is the first study in Türkiye that evaluated the vaccine safety among individuals who were infected with COVID-19 but asymptomatic or mildly symptomatic at the time of vaccination and showed the safety of this application comparatively to the non-infected individuals. Besides these favorable outcomes, it also has some limitations. First, it was based on the aggregated data from the previous phase III clinical trials of TURKOVAC, which raises concerns about selection bias of participants included in the trials following strict inclusion criteria. Although this may be accounted as a potential bias, it also provided aggregating standardized data, which increased the generalizability of the results. Second, the comparator in both phase III trials was CoronaVac, thus limiting the generalization of the results for other mRNA vaccines administered in Türkiye. Third, the analyses were kept descriptive per the primary aim of this study, which might be limited interpretation in real-world scenarios. Last but not least, the design of this study, as a secondary analysis of previous data, limited the assessment of causality between the AEFIs and the vaccines rather than possible tertiary factors, such as infections, etc. Another drawback is the overlap between adverse events evaluated in the study and COVID-19 symptoms. The higher frequency of AEFIs in the PCR(+) group is more-or-less expected, regardless of vaccination, and it should be considered that separating vaccine reactivity from infection symptoms is difficult.

## 5. Conclusions

In the seventh year of the COVID-19 pandemic, with the epidemiological characteristics shifting towards a widespread seasonal setting, more individuals will be presented with asymptomatic or mildly symptomatic infections for vaccination. Our results showed that TURKOVAC is safe to administer in these patients, and potential AEFIs that can develop are mild, tolerable, tend to be local, and can be managed easily. Although AEFIs were more frequent in the PCR(+) group, they were easily manageable. Our results support the suggestion that routine PCR testing prior to vaccination may not be warranted solely to mitigate concerns about anticipated adverse events.

## Figures and Tables

**Table 1:** Demographic characteristics of participants in the vaccine and PCR positivity groups.

	TURKOVAC			CoronaVac		
	N = 4267			N = 940		
	PCR(−)	PCR(+)	*p*	PCR(−)	PCR(+)	*p*
	*n* = 3346	*n* = 921		*n* = 675	*n* = 265	
	Mean ± SD	Mean ± SD		Mean ± SD	Mean ± SD	
Age (year)	43 ± 9	41 ± 9	<0.001	41 ± 10	39 ± 9	0.007
	***n* (%)**	***n* (%)**	***p***	***n* (%)**	***n* (%)**	***p***
Sex			0.001			0.290
Female	938 (28.0)	309 (33.6)		230 (34.1)	100 (37.7)	
Male	2408 (72.0)	612 (66.4)		445 (65.9)	165 (62.3)	

**Table 2:** Local and systemic adverse events following immunization (AEFI) in vaccine groups and both groups combined.

	TURKOVAC (N = 4267)	CoronaVac (N = 940)	Total (N = 5207)
	*n* (%)	*n* (%)	*n* (%)
Local			
Pain in injection site	849 (19.9)	112 (11.9)	961 (18.5)
Grade 1	825 (19.3)	110 (11.7)	935 (18.0)
Grade 2	24 (0.6)	2 (0.2)	26 (0.5)
Grade 3	3 (0.1)	0 (0.0)	3 (0.1)
Grade 4			
Grade 5			
Pain in arm	404 (9.5)	42 (4.5)	446 (8.6)
Grade 1	386 (9.0)	40 (4.3)	426 (8.2)
Grade 2	18 (0.4)	1 (0.1)	19 (0.4)
Grade 3	1 (0.0)	1 (0.1)	2 (0.0)
Grade 4			
Grade 5			
Swelling/nodule in injection site	222 (5.2)	22 (2.3)	244 (4.7)
Grade 1	219 (5.1)	22 (2.3)	241 (4.6)
Grade 2	3 (0.1)	0 (0.0)	3 (0.1)
Grade 3			
Grade 4			
Grade 5			
Erythema in injection site	58 (1.4)	9 (1)	67 (1.3)
Grade 1	56 (1.3)	9 (1.0)	65 (1.2)
Grade 2	2 (0.0)	0 (0.0)	2 (0.0)
Grade 3			
Grade 4			
Grade 5			
Myalgia	23 (0.5)	2 (0.2)	25 (0.5)
Grade 1	22 (0.5)	2 (0.2)	24 (0.5)
Grade 2			
Grade 3	1 (0.0)	(0.0)	1 (0.0)
Grade 4			
Grade 5			
Systemic			
Headache	451 (10.6)	126 (13.4)	577 (11.1)
Grade 1	403 (9.4)	106 (11.3)	509 (9.8)
Grade 2	50 (1.2)	24 (2.6)	74 (1.4)
Grade 3	4 (0.1)	1 (0.1)	5 (0.1)
Grade 4			
Grade 5			
Fatigue	416 (9.7)	114 (12.1)	530 (10.2)
Grade 1	393 (9.2)	105 (11.2)	498 (9.6)
Grade 2	27 (0.6)	11 (1.2)	38 (0.7)
Grade 3	1 (0.0)	1 (0.1)	2 (0.0)
Grade 4			
Grade 5			
Myalgia	370 (8.7)	76 (8.1)	446 (8.6)
Grade 1	346 (8.1)	66 (7.0)	412 (7.9)
Grade 2	33 (0.8)	10 (1.1)	43 (0.8)
Grade 3	1 (0.0)	(0.0)	1 (0.0)
Grade 4			
Grade 5			
Sore throat	367 (8.6)	99 (10.5)	466 (8.9)
Grade 1	333 (7.8)	80 (8.5)	413 (7.9)
Grade 2	43 (1.0)	21 (2.2)	64 (1.2)
Grade 3	1 (0.0)	2 (0.2)	3 (0.1)
Grade 4			
Grade 5			
Cough	320 (7.5)	91 (9.7)	411 (7.9)
Grade 1	289 (6.8)	74 (7.9)	363 (7.0)
Grade 2	38 (0.9)	17 (1.8)	55 (1.1)
Grade 3			
Grade 4			
Grade 5			

**Table 3:** Local and systemic adverse events following immunization (AEFIs) in PCR-positive and PCR-negative groups.

	PCR(+)	PCR(−)	*p* ^1^
	*n* (%)	*n* (%)	
Local			
Pain in injection site	290 (24.5)	671 (16.7)	<0.001
Pain in arm	124 (10.5)	322 (8.0)	0.008
Swelling/nodule in injection site	56 (4.7)	188 (4.7)	0.947
Erythema in injection site	20 (1.7)	47 (1.2)	0.165
Myalgia	4 (0.3)	21 (0.5)	0.418
Total ^2^	290 (24.5)	671 (16.7)	<0.001
Systemic			
Headache	176 (14.8)	401 (10.0)	<0.001
Fatigue	172 (14.5)	358 (8.9)	<0.001
Myalgia	146 (12.3)	300 (7.5)	<0.001
Sore throat	141 (11.9)	325 (8.1)	<0.001
Cough	132 (11.1)	279 (6.9)	<0.001
Total ^2^	177 (14.9)	401 (10.0)	<0.001

^1^ Chi-square; ^2^ total AEFIs were calculated case-wise and mutually exclusive, where multiple AEFIs were summarized to the binary state of present/absent.

**Table 4:** Local and systemic adverse events following immunization (AEFIs) based on PCR positivity across different time points.

	PCR(+)	PCR(−)	*p*
	*n* (%)	*n* (%)	
30 min	9 (0.8)	24 (0.6)	0.537
Local	6 (0.5)	16 (0.4)	0.614
Systemic	3 (0.3)	9 (0.2)	0.854
24 h	300 (25.3)	698 (17.4)	<0.001
Local	247 (20.8)	591 (14.7)	<0.001
Systemic	88 (7.4)	203 (5.0)	0.002
3 d	546 (46.0)	1319 (32.8)	<0.001
Local	423 (35.7)	1052 (26.2)	<0.001
Systemic	224 (18.9)	495 (12.3)	<0.001
7 d	589 (49.7)	1437 (35.7)	<0.001
Local	434 (36.6)	1082 (26.9)	<0.001
Systemic	283 (23.9)	642 (16.0)	<0.001

**Table 5:** Factors influencing the occurrence of AEFIs–logistic regression analysis.

	OR (95% CI)	*p*
Overall AEFIs		
Sex (Female)	1.42 (1.25–1.60)	<0.001
Age	0.98 (0.97–0.99)	<0.001
PCR-positive	1.87 (1.63–2.14)	<0.001
Vaccine-TURKOVAC	1.38 (1.20–1.60)	<0.001
Local AEFIs		
Sex (Female)	1.35 (1.18–1.55)	<0.001
Age	0.98 (0.97–0.98)	<0.001
PCR-positive	1.35 (1.16–1.56)	<0.001
Vaccine-TURKOVAC	3.10 (2.51–3.82)	<0.001
Systemic AEFIa		
Sex (Female)	1.16 (1.01–1.33)	0.031
Age	1.00 (0.99–1.01)	0.722
PCR-positive	1.62 (1.40–1.87)	<0.001
Vaccine-TURKOVAC	0.62 (0.54–0.73)	<0.001

## Data Availability

Proposals should be submitted to the sponsor, Health Institutes of Türkiye, who holds all the financial and intellectual rights of the study and the study vaccine TURKOVAC.

## Supplementary Materials

The following supporting information can be downloaded at https://www.mdpi.com/article/10.3390/vaccines14080657/s1, Table S1. Local and systemic adverse events of two studies for which we used pooled data; Table S2. Local and systemic adverse events following immunization (AEFIs) in PCR-positive and PCR-negative groups comparatively in vaccine groups; Table S3. Local and systemic adverse events following immunization (AEFIs) based on PCR positivity across different time points comparatively in vaccine groups.

## Abbreviations

The following abbreviations are used in this manuscript:

AEFI	Adverse events following immunization
SARS-CoV-2	Severe Acute Respiratory Syndrome Coronavirus 2
WHO	World Health Organization
CDC	Centers for Disease Control and Prevention
FDA	Food and Drug Administration
SAGE	Strategic Advisory Group of Experts on Immunization
