# Supplementary material for: Adverse Events in Following Immunization with Inactivated SARS-CoV-2 Vaccines (TURKOVAC and CoronaVac) in PCR-Positive Asymptomatic or Mildly Symptomatic Individuals Compared to PCR-Negative Recipients
Source: Vaccines (Basel). 2026 Jul 27;14(8):657. doi: 10.3390/vaccines14080657 (PMC13517549; doi:10.3390/vaccines14080657)
Supplement: Supplementary file 1 [file vaccines-14-00657-s001.zip › vaccines-4408405-supplementary.pdf]

Table S1. Local and systemic adverse events of two studies for which we used pooled data

Adverse Event	Primary Series Trial (NCT04942405)	Booster Trial (NCT05077176)	p
**Local**	32.9%	28.9%	
Pain in injection site	30.1%	16.0%	<0.001
Pain in arm	2.0%	10.0%	<0.001
Swelling/nodule in injection site	2.7%	5.1%	<0.001
Erythema in injection site	1.0%	1.4%	0.184
Myalgia	0.1%	0.6%	0.046
**Systemic**	33.6%	23.6%	
Headache	17.8%	9.6%	<0.001
Fatigue	17.3%	8.7%	<0.001
Myalgia	10.4%	8.2%	0.496
Sore throat	13.2%	8.0%	0.005
Cough	11.4%	7.2%	0.019

Table S2. Local and systemic adverse events following immunization (AEFIs) in PCR positive and PCR negative groups comparatively in vaccine groups

	TURKOVAC			CoronaVac		
	PCR(+) n (%)	PCR(−) n (%)	p[1]	PCR(+) n (%)	PCR(−) n (%)	p[1]
**Local**						
Pain in injection site	248 (26.9)	601 (18.0)	<0.001	42 (15.8)	70 (10.4)	0.020
Pain in arm	112 (12.2)	292 (8.7)	0.002	12 (4.5)	30 (4.4)	0.955
Swelling/nodule in injection site	52 (5.6)	170 (5.1)	0.494	4 (1.5)	18 (2.7)	0.291
Erythema in injection site	18 (2.0)	40 (1.2)	0.078	2 (0.8)	7 (1.0)	1.000 [2]
Myalgia	4 (0.4)	19 (0.6)	0.801 [2]	0 (0.0)	2 (0.3)	-
Total [3]	248 (26.9)	601 (18.0)	<0.001	42 (15.8)	70 (10.4)	0.020
**Systemic**						
Headache	119 (12.9)	332 (9.9)	0.009	57 (21.5)	69 (10.2)	<0.001
Fatigue	120 (13.0)	296 (8.8)	<0.001	52 (19.6)	62 (9.2)	<0.001
Myalgia	107 (11.6)	263 (7.9)	<0.001	39 (14.7)	37 (5.5)	<0.001
Sore throat	100 (10.9)	267 (8.0)	0.006	41 (15.5)	58 (8.6)	0.002
Cough	94 (10.2)	226 (6.8)	<0.001	38 (14.3)	53 (7.9)	0.002
Total [3]	120 (13.0)	332 (9.9)	0.007	57 (21.5)	69 (10.2)	<0.001

[1] Chi-square; [2] Fisher's Exact Test; [3] Total AEFIs were calculated case-wise and mutually exclusive. where multiple AEFIs were summarized to the binary state of present/absent.

Table S3. Local and systemic adverse events following immunization (AEFIs) based on PCR positivity across different time points comparatively in vaccine groups

	**PCR(+)**			**PCR(−)**		
	**TURKOVAC N=921**	**CoronaVac N=265**	**p**	**TURKOVAC N=3346**	**CoronaVac N=675**	**p**
	**n (%)**	**n (%)**		**n (%)**	**n (%)**	
**30 min**	7 (0.8)	2 (0.8)	0.69	15 (0.4)	9 (1.3)	0.014
Local	5 (0.5)	1 (0.4)	0.88	9 (0.3)	7 (1.0)	0.011
Systemic	2 (0.2)	1 (0.4)	-	7 (0.2)	2 (0.3)	0.99
**24 h**	232 (25.2)	68 (25.7)	0.94	594 (17.8)	104 (15.4)	0.16
Local	208 (22.6)	39 (14.7)	0.007	512 (15.3)	79 (11.7)	0.019
Systemic	50 (5.4)	38 (14.3)	<0.001	163 (4.9)	40 (5.9)	0.300
**3 d**	440 (47.8)	106 (40.0)	0.03	1149 (34.3)	170 (25.2)	<0.001
Local	373 (40.5)	50 (18.9)	<0.001	944 (28.2)	108 (16)	<0.001
Systemic	150 (16.3)	74 (27.9)	<0.001	408 (12.2)	87 (12.9)	0.66
**7 d**	470 (51.0)	119 (44.9)	0.091	1243 (37.1)	194 (28.7)	<0.001
Local	381 (41.4)	53 (20.0)	<0.001	968 (28.9)	114 (16.9)	<0.001
Systemic	196 (21.3)	87 (32.8)	<0.001	525 (15.7)	117 (17.3)	0.32
